# The Natural Agonist of Estrogen Receptor β Silibinin Plays an Immunosuppressive Role Representing a Potential Therapeutic Tool in Rheumatoid Arthritis

**DOI:** 10.3389/fimmu.2018.01903

**Published:** 2018-08-17

**Authors:** Maria Luisa Dupuis, Fabrizio Conti, Angela Maselli, Maria Teresa Pagano, Anna Ruggieri, Simona Anticoli, Alessandra Fragale, Lucia Gabriele, Maria Cristina Gagliardi, Massimo Sanchez, Fulvia Ceccarelli, Cristiano Alessandri, Guido Valesini, Elena Ortona, Marina Pierdominici

**Affiliations:** ^1^Center for Gender Specific Medicine, Istituto Superiore di Sanità, Rome, Italy; ^2^Rheumatology Unit, Department of Internal Medicine and Medical Specialties, Sapienza University of Rome, Rome, Italy; ^3^Department of Oncology and Molecular Medicine, Istituto Superiore di Sanità, Rome, Italy; ^4^Core Facilities, Istituto Superiore di Sanità, Rome, Italy

**Keywords:** estrogen receptor β, silibinin, T lymphocytes, immunity, sex, rheumatoid arthritis

## Abstract

Estrogens, in particular 17β-estradiol (E2), have a strong influence on the immune system and also affect pathological conditions such as autoimmune diseases. The biological effects of E2 are mediated by two intracellular receptors, i.e., estrogen receptor (ER)α and ERβ, which function as ligand-activated nuclear transcription factors producing genomic effects. Immune cells express both ERα and ERβ that play a complex role in modulating inflammation. Phytoestrogens display estrogen-like effects. Among them, silibinin, the major active constituent of silymarin extracted by the milk thistle (*Silybum marianum*), has been suggested to have an ERβ selective binding. Silibinin is known to have anti-inflammatory, hepatoprotective, and anticarcinogenic effects; however, the role of silibinin in modulating human immune responses and its impact on autoimmunity remains unclear. Aim of this study was to dissect the ability of the ERβ natural ligand silibinin to modulate T cell immunity, taking into account possible differences between females and males, and to define its possible role as therapeutic tool in immune-mediated diseases. To this purpose, female and age-matched male healthy subjects and patients with active rheumatoid arthritis (RA) were recruited. We evaluated the ability of silibinin to modulate ERβ expression in T lymphocytes and its effects on T cell functions (i.e., apoptosis, proliferation, and cytokine production). We also analyzed whether silibinin was able to modulate the expression of microRNA-155 (miR-155), which strongly contributes to the pathogenesis of RA driving aberrant activation of the immune system. We demonstrated that silibinin upregulated ERβ expression, induced apoptosis, inhibited proliferation, and reduced expression of the pro-inflammatory cytokines IL-17 and TNF-α, through ERβ binding, in T lymphocytes from female and male healthy donors. We obtained similar results in T lymphocytes from patients with active RA in term of apoptosis, proliferation, and cytokine production. In addition, we found that silibinin acted as an epigenetic modifier, down-modulating the expression of miR-155. In conclusion, our data demonstrated an immunosuppressive role of silibinin, supporting its application in the treatment of autoimmune diseases as drug, but also as dietary nutritional supplement, opening new perspective in the field of autoimmune disease management.

## Introduction

It is well known that estrogen (17β-estradiol—E2) influences different aspects of the immune system function and potentially affects the risk, activity, and progression of autoimmune diseases (1–4). In particular, E2 is able to modulate different aspects of immune responses, e.g., lymphocyte proliferation and apoptosis, cytokine, or antibody production (5–8). The immunomodulatory effects exerted by E2 are, at least partially, responsible for the existing differences between female and male immune systems, with females mounting stronger humoral and cellular immune responses than males (6). As a consequence, females are generally more resistant to infection but more susceptible to autoimmune diseases that are typically dominant in women in comparison to men (4, 9, 10). The most important factors responsible for this sex bias are sex hormones, genetic and epigenetic factors, as well as sociological differences between genders. Notably autoimmune diseases differ between males and females not only for their incidence but also for clinical outcome and response to therapy.

17β-estradiol effects are mediated by two intracellular estrogen receptors (ER), i.e., ERα and ERβ, which act as ligand-activated nuclear transcription factors generating genomic effects (8, 11). Our and other groups have demonstrated that immune cells express both ERα and ERβ (12–15) which have a complex role in modulating inflammation, thus representing potential therapeutic targets in autoimmune diseases (16–20). In particular, the low intracellular expression level of ERβ has been demonstrated to be associated with high disease activity in chronic inflammatory diseases such as systemic lupus erythematosus (21) and inflammatory bowel diseases (22). The downregulation of this receptor has been found to be dependent by a pro-inflammatory microenvironment (22). Accordingly, ERβ agonist ligands have been suggested to dampen inflammation in animal models of autoimmune diseases (17, 23).

Interestingly, some phytoestrogens, naturally occurring plant compounds, display ERβ selective binding with estrogen-like effects. Among them, silibinin (24), the major active constituent of silymarin extracted by the milk thistle (*Silybum marianum*), has been suggested by *in silico* studies to have an ERβ selective binding acting as agonist of this ER (25). Silibinin has been demonstrated to have anti-inflammatory, hepatoprotective, and anticarcinogenic properties interfering with multiple biochemical pathways (26); however, the role of silibinin in modulating human immune responses and its impact on autoimmunity remains unclear.

Hence, the aim of this study was to dissect the ability of the ERβ natural ligand silibinin to modulate T cell immunity, taking into account possible differences between females and males, and to define its possible role as a therapeutic tool in immune-mediated diseases. To this purpose, we first evaluated the ability of silibinin to modulate ERβ expression in T lymphocytes from female and age-matched male healthy subjects and its effects on T cell functions (i.e., apoptosis, proliferation, and cytokine production). Then we analyzed the effects played by silibinin on T lymphocytes from patients affected by rheumatoid arthritis (RA), a chronic autoimmune inflammatory disease, characterized by synovial inflammation and by cartilage and bone destruction (27), in which T lymphocytes play a key pathogenetic role (27). We also evaluated whether silibinin could modulate the expression of microRNA-155 (miR-155) which is involved in the modulation of T lymphocyte immunity (28) and strongly contributes to the pathogenesis of RA driving aberrant activation of the immune system (29–31).

## Materials and Methods

### Study Population

Forty-four healthy subjects (23 postmenopausal females and 21 age-matched males, age range 55–75 years) as well as 10 postmenopausal female patients and 4 age-matched male patients with active RA, who had an inadequate response to drugs, followed at the Rheumatology outpatient Clinic (Arthritis Center, Policlinico Umberto I, Sapienza University of Rome, Italy), were included in the study. All RA patients fulfilled the 2010 American College of Rheumatology/European League against Rheumatism (ACR/EULAR) classification criteria (32). Exclusion criteria were pregnancy, treatment with any kind of hormones. Clinical evaluation included the count of swollen and tender joints, patient and physician global disease assessment by VAS (0–100 mm). Disease activity was measured by Disease Activity Score 28 (DAS28) and clinical response was evaluated according to EULAR response criteria (33). The following laboratory tests were performed: complete blood count, erythrocyte sedimentation rate, C-reactive protein, antinuclear antibodies, rheumatoid factor, and anti-cyclic citrullinated peptide antibodies. Demographic and clinical features of RA patients are shown in Table 1.

**Table 1:** Demographic and clinical features of rheumatoid arthritis patients (females, *N* = 10; males, *N* = 4).

Patients features	Females	Males
Median age (years), IQR	60 (18.5)	61.5 (9.0)
Median disease duration (years), IQR	17 (15.5)	9.5 (5.5)
Median DAS28, IQR	5.5 (1.3)	5.9 (1)
PDN, *N*/%	1/88.8	1/25
sDMARDs treatment, *N*/%	5/62.5	4/100
bDMARDs drugs, *N*/%	4/44.4	0

*IQR, InterQuartile range; PDN, prednisone; DAS28, Disease Activity Score 28; sDMARDs, synthetic disease-modifying antirheumatic drugs; bDMARDs, biologic disease-modifying antirheumatic drugs; N, number*.

This study was carried out in accordance with the recommendations of the Declaration of Helsinki. Written informed consent was obtained from all subjects, and the ethics committee of the Policlinico Umberto I (Rome, Italy) approved the study.

### Isolation of Peripheral Blood Mononuclear Cells (PBMCs) and Cell Culture Conditions

Peripheral blood mononuclear cells were isolated by Ficoll-Hypaque density-gradient centrifugation and cultured in RPMI-1640 medium without phenol red (Gibco BRL, Grand Island, NY, USA) supplemented with 10% charcoal-stripped fetal bovine serum (Hyclone Laboratories, South Logan, UT, USA), 2 mM glutamine (Sigma, St. Louis, MO, USA), and 50 µg/ml gentamycin (Sigma). Silibinin (Sigma) was dissolved in dimethyl sulfoxide and diluted in RPMI 1640. Preliminary dose response and time course experiments showed that silibinin should be used at a dose of 50 µM and at 24–72 h of culture (depending on the studied parameters) to obtain the highest detectable changes in the absence of toxic effects. For lymphocyte activation, PBMCs or sorted CD4^+^CD45RA^−^CCR6^+^CXCR3^−^ (see below for sorting) were cultured in the presence of plate-bound anti-CD3 monoclonal antibody (mAb, clone UCHT1, R&D Systems, Minneapolis, MN, USA) at 4 µg/ml for 72 h and treated with silibinin for the last 48 h of culture. In separate experiments, cells were pretreated with 100 nM 4-[2-phenyl-5,7-bis(trifluoromethyl)pyrazolo[1,5-*a*]pyrimidin-3-yl]phenol (PHTPP) ERβ antagonist (Tocris Cookson, Ellisville, MO, USA) for 1 h before adding silibinin.

For cytokine production, untreated or treated PBMCs were stimulated as follows: (i) for IFN-γ, TNF-α, IL-2, and IL-4 analysis, 25 ng/ml phorbol myristate acetate (PMA, Sigma) and 1 µg/ml ionomycin (Sigma) for the last 16 h of culture; (ii) for IL-17 analysis, 50 ng/ml PMA (Sigma) and 1 µg/ml ionomycin (Sigma) for the last 4 h of culture; and (iii) for IL-10, 2.5 µg/ml phytohemagglutinin (Sigma) for the last 16 h of culture. To inhibit cytokine secretion, 10 µg/ml brefeldin A (Sigma) was added to each condition at the beginning of stimulation.

### Flow Cytometry

Cell surface phenotyping was performed by flow cytometry as previously described (13). Allophycocyanin (APC)-conjugated anti-CD3, APC- or phycoerythrin (PE)-conjugated anti-CD4, peridinin chlorophyll protein-conjugated anti-CD8 mAbs (all from BD Biosciences, San Jose, CA, USA) were used. Equal amount of mouse IgG isotype control was run in parallel. Analysis of cytokine production at the single cell level was performed as previously described with minor changes (34). Briefly, treated cells (see above for details) were either fixed with 4% paraformaldehyde and permeabilized with FACS permeabilizing solution (BD Biosciences) for IFN-γ, TNF-α, IL-2, IL-4, and IL-10 detection or fixed and permeabilized with intracellular fixation and permeabilization buffer (eBioscience, San Diego, CA, USA) for IL-17 detection. The following cytokine-specific mAbs were used: fluorescein isothiocyanate (FITC)-labeled anti-IFN-γ, FITC-labeled anti-IL-2, PE-labeled anti-TNF-α, PE-labeled anti-IL-4, PE-labeled anti-IL-10 (all from BD Biosciences), and FITC-labeled anti-IL-17A (eBioscience). Appropriate isotypic negative controls were run in parallel. Apoptosis was quantified using FITC- or PE-conjugated annexin V (AV) and propidium iodide (PI) detection kit (Marine Biological Laboratory, Woods Hole, MA, USA) according to the manufacturer’s protocol. Proliferation was evaluated by measuring Ki-67 nuclear antigen expression using FITC-labeled anti-human Ki-67 mAb according to the manufacturer’s protocol (BD Biosciences). For ERβ intracellular staining of sorted CD4^+^CD45RA^−^CCR6^+^CXCR3^−^ Th17 lymphocytes, cells were fixed and permeabilized as described above, and stained with the anti-ERβ mAb (clone CWK-F12 from DSHB, Iowa City, IA, USA). Equal amount of mouse IgG isotype control was run in parallel. The primary antibody was visualized by FITC-conjugated F(ab′)2 fragment secondary antibody (Abcam, Cambridge, UK).

To determine the frequency of T cell subsets, total lymphocytes were first gated by forward and side scatter and then additionally gated for CD3 and CD4 or CD3 and CD8 molecule expression. Acquisition was performed on a FACSCalibur flow cytometer (BD Biosciences) and at least 50,000 events per sample were run. Data were analyzed using the Cell Quest Pro software (BD Biosciences).

### MACS and FACS Cell Sorting

For Western blot and quantitative real-time PCR (qRT-PCR) analyses, untouched T cells were separated using the Pan T Cell isolation Kit II (Miltenyi Biotec, Bergisch-Gladbach, Germany). The purity of recovered cells, assessed by flow cytometer, was ≥97%.

For apoptosis and ERβ analyses of Th17 cells (i.e., CD4^+^CD45RA^−^CCR6^+^CXCR3^−^), CD4^+^ T cells were separated from PBMC by positive selection using CD4 MicroBeads (Miltenyi Biotec), with a purity ≥97%, as determined by flow cytometer. Then, CD4^+^CD45RA^−^CCR6^+^CXCR3^−^ T cell subset was sorted by FACS (BD FACSAria; BD Biosciences) upon staining with the following mixture of mAb: CD4 PE/Cy7 (BD Biosciences), CD45RA FITC (BD Biosciences), CCR6 PE (Miltenyi Biotec), and CXCR3 APC (BD Biosciences). Sorted T cell subset was on average >95% pure as determined by postsorting flow cytometry analysis.

### SDS-PAGE and Western Blot

SDS-PAGE and Western blot were performed as previously described (13). Briefly, cells were lysed in RIPA buffer [100 mM tris(hydroxymethyl)aminomethane (Tris)–HCl pH 8, 150 mM NaCl, 1% Triton X-100, 1 mM MgCl_2_] in the presence of a complete protease-inhibitor mixture. Protein content was determined by the Bradford assay (Bio-Rad Laboratories, Richmond, CA, USA). Cell lysates (30 µg/ml) were loaded onto SDS-PAGE and, after electrophoresis, proteins were transferred onto nitrocellulose membrane (GE Healthcare, Pittsburgh, PA, USA) by means of a Trans-Blot transfer cell (Bio-Rad Laboratories). The membranes were then blocked in 5% nonfat milk and incubated with the appropriate antibodies in Tris-buffered saline containing 0.1% Tween 20 and 5% nonfat milk. Anti-ERβ mAb (clone CWK-F12 from DSHB) was used as primary Ab. Peroxidase-conjugated goat anti-mouse IgG was used as secondary Ab (Bio-Rad Laboratories) and the reactions were developed using the SuperSignal West Pico Chemiluminescent Substrate (Pierce, Rockford, IL, USA). To ensure the presence of equal amounts of protein, the membranes were reprobed with a rabbit anti-human glyceraldehyde 3-phosphate dehydrogenase Ab (Sigma). Quantification of protein expression was performed by densitometry analysis of the autoradiograms (GS-700 Imaging Densitometer, Bio-Rad Laboratories).

### qRT-PCR Analysis of miR-155 Expression Level

Total RNA, including short RNA, was isolated from T lymphocytes of female RA patients, treated and untreated with silibinin for 48 h, using the Total RNA Purification Plus Kit (Norgen Biotek Corp., Thorold, ON, Canada), according to the manufacturer’s instructions. RNA samples, after quantity evaluation using a NanoDrop ND-1000 spectrophotometer, were used for qRT-PCR analysis. miR-155 and RNU6B, as normalizator, expression levels were quantitated using specific inventoried TaqMan MicroRNA Assays (Thermo Fisher Scientific, Waltham, MA USA), according to the manufacturer’s instructions, and all samples were run in triplicate. Briefly, 15 ng of each RNA sample were reverse transcribed by the Taq-Man^®^ MicroRNA Reverse Transcription (RT) Kit (Thermo Fisher Scientific) using individual miR-specific RT primers, and 1.3 µl of RT product were analyzed by qRT-PCR on the ABI7000 Real-Time PCR System (Applied Biosystem, Foster City, CA, USA). The relative expression level of miR-155 was determined by the 2−ΔΔCt method, after normalization to the RNU6B Ct. 1.5 miR fold changes between RA patients treated or untreated with silibinin were considered significant.

### qRT-PCR Analysis of ERβ mRNA Expression Level

Total RNA was extracted from cells using the RNeasy Mini kit (Qiagen, Milan, Italy). RNA was DNase-I digested (Roche) and reverse transcribed as previously described (35). Quantitative PCR was performed in duplicate by the real-time fluorescence detection method with the fluorescent DNA binding dye SYBR green (Power SYBR Green PCR master kit; Applied Biosystems) by using an ABI PRISM 7900 (Applied Biosystems). The relative expression levels were calculated by the comparative cycle threshold (ΔΔCt) method and were normalized by hypoxanthine-guanine phosphoribosyl transferase expression. Homo sapiens estrogen receptor 2 (ESR2, ERβ1) primers used for RT-PCR were designed by using the Primer3Plus software, crossing exon-intron junctions and checking for secondary structures; sequences are 5′-GCTCCTGTCCCACGTCAG-3′, 5′-CACATAATCCCATCCCAAGC-3′.

### ERβ Silencing by Small Interfering RNA (siRNA)

The silencing of ERβ was performed with the following Silencer Select siRNA for ESR2, sense, AGUGUACAAUCGAUAAAAATT, antisense, UUUUUAUCGAUUGUACACUGA (Ambion, Milan, Italy). Silencer select negative control siRNA (SSNC, Ambion) was also used as negative control. For transfection of human T lymphocyte, the Amaxa Human T cell Nucleofector^®^ kit was used (Lonza, Walkersville, MD, USA) according to the manufacturer’s protocol. In brief, 6 × 10^6^ cells per condition were resuspended in 100 µl of the Nucleofactor kit solution, combined with 300 nM of the indicated siRNA or pmaxGFP vector (2 µg), and electroporated using the U-014 program of the Nucleofector (Amaxa Biosystems, Köln, Germany). Transfection efficiency was monitored in all samples by FACS analysis of GFP fluorescence and was about 50%. Cell apoptosis, measured by AV/PI detection kit was <20% (data not shown). After 6 h, siRNA-transfected PBMCs were treated with silibinin (Sigma) for 48 h and analyzed for IL-17 and TNF-α expression after stimulation with PMA and ionomycin (both from Sigma) in the presence of brefeldin A (Sigma). See above for methodological details.

### Statistical Analysis

Statistical analysis was performed by the Mann–Whitney *U* test using GraphPad Prism, version 7.0 software (GraphPad Software, San Diego, CA, USA). A *P* value <0.05 was considered statistically significant.

## Results

### The Natural ERβ Agonist Silibinin Modulates ERβ Expression in Peripheral Blood T Lymphocytes From Female and Male Healthy Subjects

**Figure 1:**
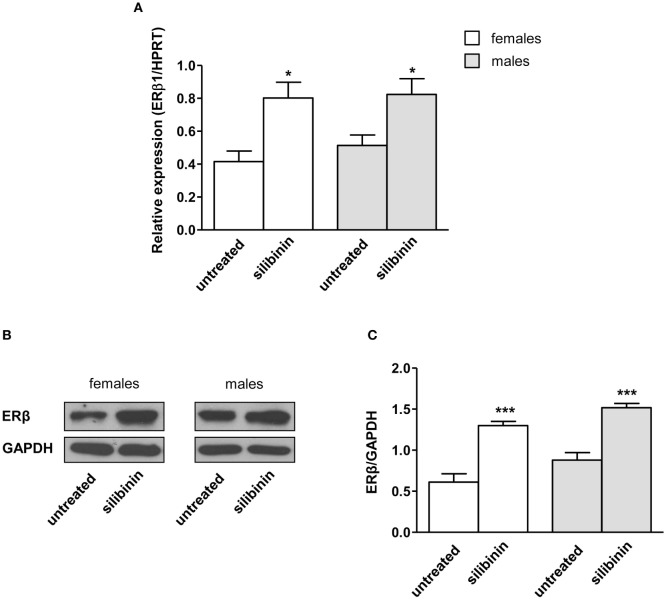
Silibinin increased the expression of estrogen receptor (ER) β in peripheral blood T lymphocytes from female and male healthy subjects. **(A)** ERβ1 mRNA levels were evaluated by quantitative real-time PCR after 24 h silibinin treatment. Data are expressed as ratios of the expression of ERβ1 and hypoxanthine-guanine phosphoribosyl transferase (HPRT) gene. Results are shown as mean ± SD from 10 randomly selected female and male healthy subjects. **(B,C)** ERβ protein levels were also evaluated by Western blot analysis of T-cell lysates after 24 h silibinin treatment. Blots shown are representative of experiments performed in T cells from 10 randomly selected female and male healthy subjects **(B)**. Densitometry analysis of ERβ levels relative to glyceraldehyde 3-phosphate dehydrogenase (GAPDH) is also shown. Values are expressed as mean ± SD **(C)**. *P < 0.05, ***P < 0.001 versus untreated cells.

As stated above, we and other groups previously demonstrated that immune cells have detectable levels of intracellular ERβ (12–14). As literature data indicate for phytoestrogens the ability to upregulate ERβ in different cell types (36, 37), we first evaluated if this effect could be also evident in silibinin-treated T lymphocytes, taking into account possible differences between females and males. To this aim, both mRNA and protein expression level of ERβ was determined by qRT-PCR and Western blot analysis in peripheral T lymphocytes (Figures 1A–C) from healthy subjects after 16 h treatment with silibinin. A significant increase of ERβ mRNA expression level was detectable after treatment with silibinin in T cells (treated versus untreated cells, *P* = 0.01 and *P* = 0.0274, in females and males, respectively, Figure 1A). Hence, we analyzed by Western blot the protein expression level of ERβ in T lymphocytes and we found significantly higher levels of ERβ in silibinin-treated cells when compared with untreated cells (treated versus untreated cells, *P* < 0.0001 in both females and males, Figures 1B,C). T lymphocytes from female and male subjects showed comparable susceptibility to silibinin treatment.

### Silibinin-Dependent Effects on Cell Apoptosis, Cell Proliferation, and Cytokine Production in Peripheral Blood T Lymphocytes From Female and Male Healthy Subjects

**Figure 2:**
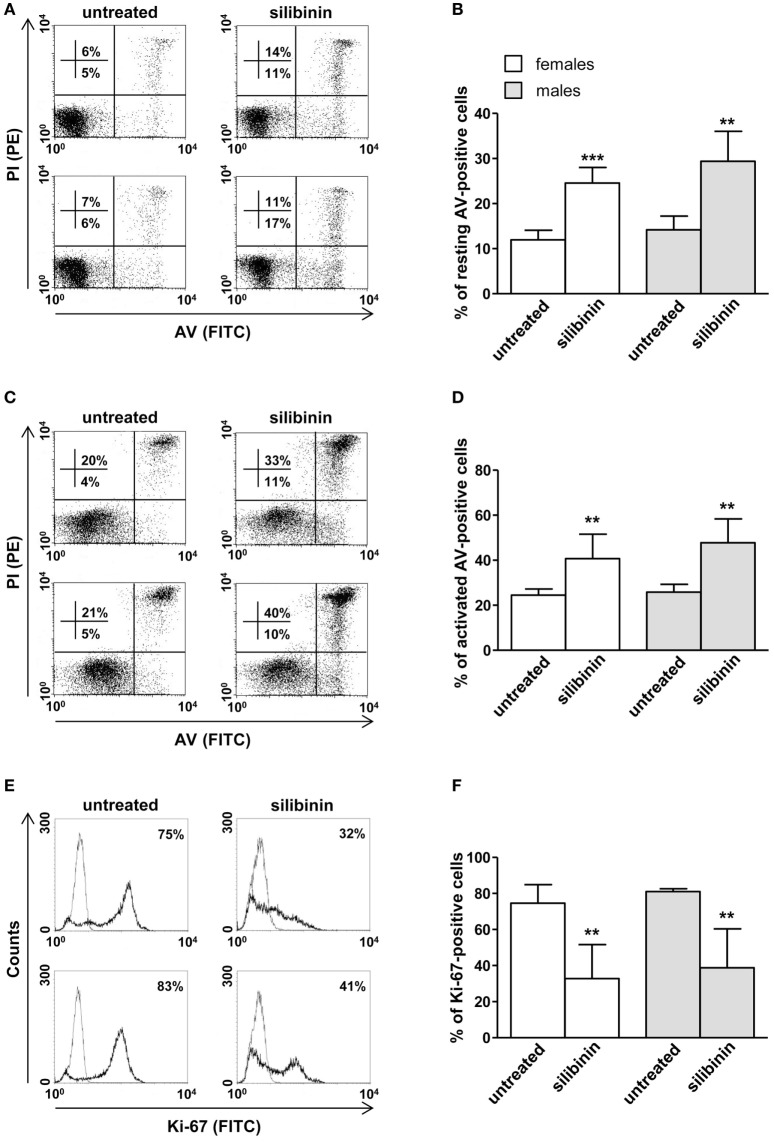
Silibinin-dependent effects on apoptosis and proliferation levels of peripheral blood T lymphocytes from female and male healthy subjects. All experiments were performed in 23 female and 21 male healthy subjects. **(A–D)** Apoptosis assay involving dual staining with annexin V (AV) and propidium iodide (PI) was carried out using flow cytometry in resting T cells treated or not with silibinin for 48 h **(A,B)** and in T cells activated by anti-CD3 monoclonal antibody (mAb) for 72 h and treated or not with silibinin for the last 48 h of culture **(C,D)**. Results from representative female (upper panels) and male (lower panels) healthy donors are shown **(A,C)**. Numbers reported represent the percentages of AV positive/PI negative (early apoptotic, bottom right quadrant) and AV positive/PI positive (late apoptotic or necrotic cells, top right quadrant). Data referred to both AV positive/PI negative and AV positive/PI positive cells are also reported as mean ± SD **(B,D)**. **(E,F)** Cell proliferation was evaluated by flow cytometry measuring Ki-67 nuclear antigen expression in T lymphocytes after activation with anti-CD3 mAb for 72 h and treatment with silibinin for the last 48 h of culture. Results from representative female (upper panels) and male (lower panels) healthy donors are shown **(E)**. Data are also reported as mean ± SD **(F)**. **P < 0.01, ***P < 0.001 versus untreated cells.

As a second step, we evaluated the ability of silibinin to impact T lymphocyte homeostasis in term of cell apoptosis, proliferation, and cytokine production. Silibinin was able to induce a significant increase in resting T lymphocyte apoptosis (treated versus untreated cells, *P* = 0.0002 and *P* = 0.0074, in females and males, respectively, Figures 2A,B). Similarly, activated T cells treated with silibinin showed a significant increase of apoptotic levels (treated versus untreated cells, *P* = 0.0029 and *P* = 0.0013, in females and males, respectively, Figures 2C,D).

In parallel, a significant reduction of proliferation of activated T lymphocytes, measured by the analysis of nuclear antigen Ki-67 expression, was observed after treatment with silibinin (treated versus untreated cells, *P* = 0.0024 and *P* = 0.0081, in females and males, respectively, Figures 2E,F).

**Figure 3:**
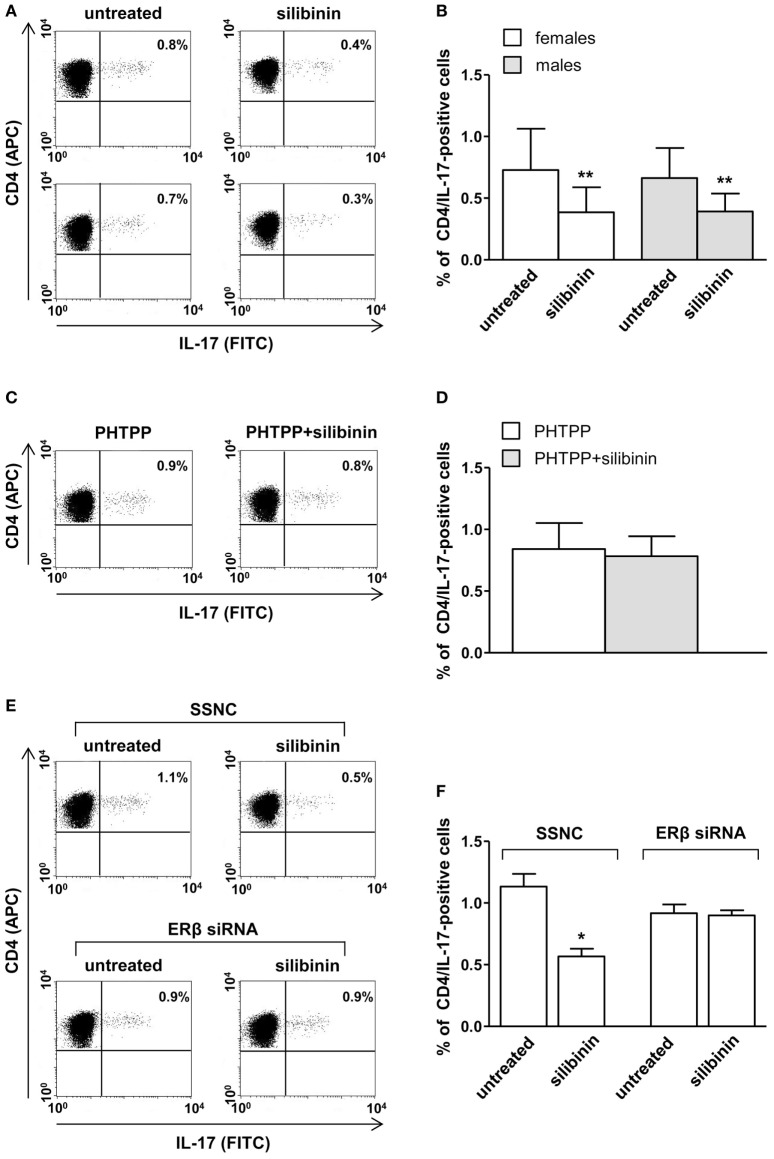
Silibinin decreased IL-17 expression in peripheral blood T lymphocytes from female and male healthy subjects acting through estrogen receptor (ER) β binding. **(A,B)** Cytokine expression was analyzed in 23 female and 21 male healthy subjects by flow cytometry after 48 h of culture with silibinin and stimulation with phorbol myristate acetate (PMA) and ionomycin in the presence of brefeldin for the last 4 h of culture as detailed in Section “Materials and Methods.” Results from representative female (upper panels) and male (lower panels) healthy donors are shown **(A)**. Data are also reported as mean ± SD **(B)**. **(C,D)** IL-17 expression was analyzed in T lymphocytes from three randomly selected female healthy donors pretreated with the ERβ antagonist PHTTP for 1 h before adding silibinin. Results from a representative female healthy donor are shown **(C)**. Data are also reported as mean ± SD **(D)**. **(E,F)** IL-17 expression was analyzed in T lymphocytes from three randomly selected female healthy donors after silencing ERβ expression. Results from a representative female healthy donor are shown **(E)**. Data are also reported as mean ± SD **(F)**. *P < 0.05, **P < 0.01 versus untreated cells. Abbreviation: SSNC, silencer select negative control siRNA.

**Figure 4:**
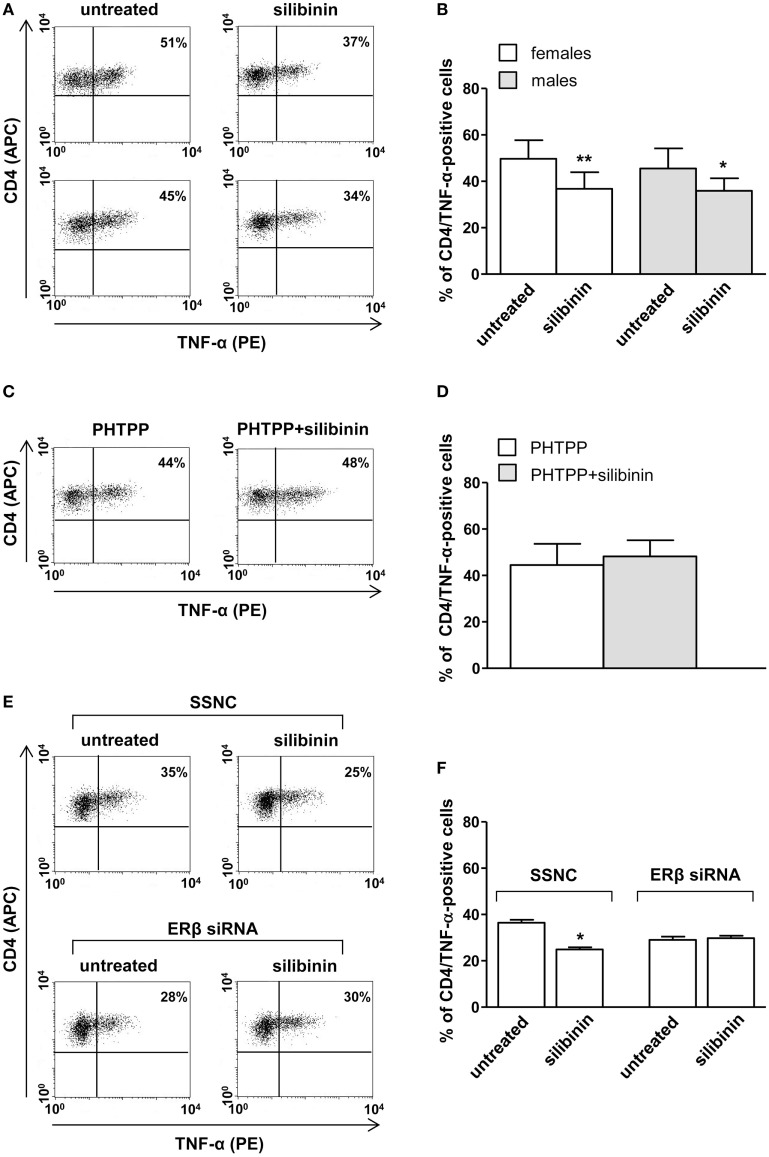
Silibinin decreased TNF-α expression in peripheral blood CD4^+^ T lymphocytes from female and male healthy subjects acting through ERβ binding. **(A,B)** Cytokine expression were analyzed in 23 female and 21 male healthy subjects by flow cytometry after 48 h of culture with silibinin and stimulation with phorbol myristate acetate (PMA) and ionomycin in the presence of brefeldin for the last 16 h of culture as detailed in Section “Materials and Methods.” Results from representative female (upper panels) and male (lower panels) healthy donors are shown **(A)**. Data are also reported as mean ± SD **(B)**. **(C,D)** TNF-α expression was analyzed in T lymphocytes from three randomly selected female healthy donors pretreated with the ERβ antagonist PHTTP for 1 h before adding silibinin. Results from a representative female healthy donor are shown **(C)**. Data are also reported as mean ± SD **(D)**. **(E,F)** TNF-α expression was analyzed in T lymphocytes from three randomly selected female healthy donors after silencing ERβ expression. Results from a representative female healthy donor are shown **(E)**. Data are also reported as mean ± SD **(F)**. *P < 0.05, **P < 0.01 versus untreated cells. Abbreviation: SSNC, silencer select negative control siRNA.

A panel of pro-inflammatory (IFNγ, TNF-α, IL-2, and IL-17) and anti-inflammatory (IL-4 and IL-10) cytokines was also studied. Notably, silibinin significantly reduced the intracellular expression level of the pro-inflammatory cytokines IL-17 and TNF-α in CD4^+^ T lymphocytes (for IL-17, treated versus untreated cells, *P* = 0.0078 and *P* = 0.0077, in females and males, respectively, Figures 3A,B; for TNF-α, treated versus untreated cells, *P* = 0.0028 and *P* = 0.0272, in females and males, respectively, Figures 4A,B). No changes were induced by silibinin in the percentage of CD8^+^/TNF-α^+^, CD4^+^/and CD8^+^/IL-2^+^, IFNγ^+^, IL-4^+^, and IL-10^+^ T lymphocytes (Table 2). In order to clarify the role of ERβ in the anti-inflammatory effects induced by silibinin, we used two different approaches: (i) the pretreatment of PBMC with the ERβ antagonist PHTTP and (ii) the silencing of ERβ with specific siRNA to knockdown ERβ gene. Notably, in both experimental conditions, silibinin lost the ability to inhibit IL-17 (Figures 3C–F) and TNF-α expression (Figures 4C–F), confirming that the observed effects were mediated by ERβ binding.

**Table 2:** Cytokine expression at the single cell level by flow cytometry analysis of CD4^+^ and CD8^+^ T lymphocytes from female and male healthy donors.

Cytokines	Females			Males		
	Untreated	Silibinin	*P*	Untreated	Silibinin	*P*
% CD4^+^/IL-17^+^	0.7 ± 0.3	0.4 ± 0.2	0.0078	0.7 ± 0.2	0.4 ± 0.1	0.0077
% CD4^+^/TNF-α^+^	50 ± 8	37 ± 7	0.0028	45 ± 9	36 ± 5	0.0272
% CD8^+^/TNF-α^+^	30 ± 12	23 ± 11	0.1022	44 ± 23	36 ± 26	0.0943
% CD4^+^/IL-2^+^	43 ± 12	39 ± 12	0.1141	49 ± 10	38 ± 19	0.0622
% CD8^+^/IL-2^+^	18 ± 6	16 ± 9	0.0562	23 ± 6	17 ± 10	0.0703
% CD4^+^/IFNγ^+^	30 ± 11	26 ± 6	0.4936	33 ± 21	42 ± 22	0.1236
% CD8^+^/IFNγ^+^	56 ± 14	48 ± 18	0.0937	65 ± 33	70 ± 28	0.7238
% CD4^+^/IL-4^+^	0.3 ± 0.2	0.2 ± 0.1	0.0979	0.7 ± 0.7	1.4 ± 1.5	0.2364
% CD8^+^/IL-4^+^	0.3 ± 0.2	0.1 ± 0.1	0.0655	0.1 ± 0.1	0.1 ± 0.1	0.9892
% CD4^+^/IL-10^+^	1.1 ± 0.6	1.1 ± 0.7	0.9644	0.9 ± 0.7	1 ± 1	0.5081

*For CD4^+^ and CD8^+^ T lymphocyte subsets, data were expressed as the percentage of each subset within the CD3^+^CD4^+^ or CD3^+^CD8^+^ population considered as 100%. Data are represented as mean ± SD from 23 female and 21 male healthy subjects. P values were calculated using the Mann–Whitney U test*.

Also in this set of experiments, no significant difference was observed between cells from male and female subjects after silibinin treatment (Figures 2–4).

To investigate whether the silibinin-mediated IL-17 inhibition could be due to elimination of memory Th17 cells or by a block of IL-17 production, we first evaluated intracellular ERβ expression in sorted Th17 cells (i.e., CD4^+^CD45RA^−^CCR6^+^CXCR3^−^, Figures S1A,B in Supplementary Material). As expected, the Th17 cell subset expressed intracellular ERβ. Then, we evaluated apoptosis level of this cell subset, treated or not with silibinin (Figure S1C in Supplementary Material). An increase of apoptotic level after silibinin treatment was detected, suggesting that the effect of silibinin on IL-17 production was, at least partially, due to apoptosis induction in this cell subset. Also in this case, the ERβ antagonist PHTTP was able to inhibit silibin-induced apoptosis.

### Silibinin Effects on Peripheral Blood T Lymphocytes From Patients With Active RA

**Figure 5:**
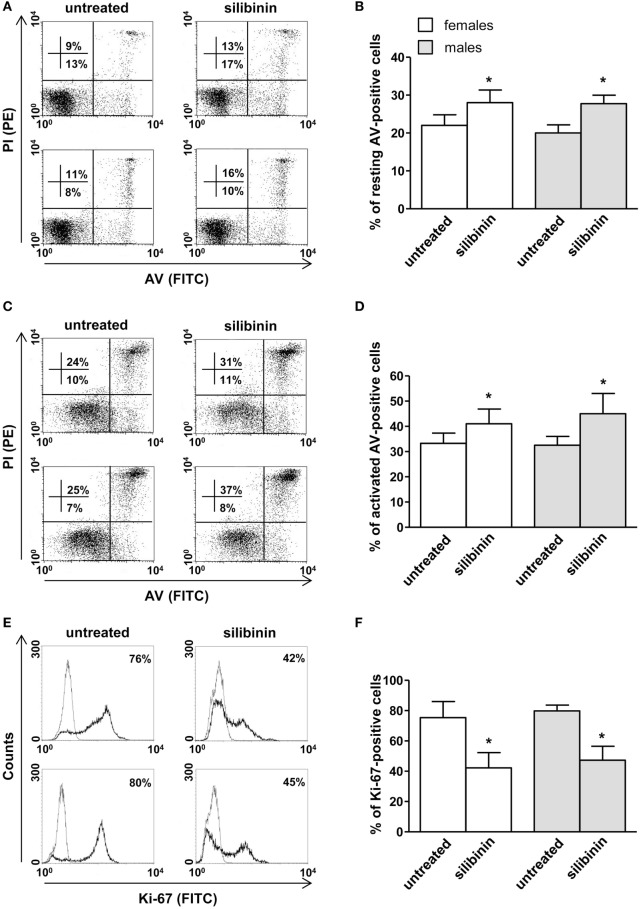
Silibinin-dependent effects on apoptosis and proliferation levels of peripheral blood T lymphocytes from patients with active rheumatoid arthritis (RA). All experiments were performed in 10 female and 4 male RA patients. **(A–D)** Apoptosis assay involving dual staining with annexin V (AV) and propidium iodide (PI) was carried out using flow cytometry in resting T cells treated or not with silibinin for 48 h **(A,B)** and in T cells activated by anti-CD3 monoclonal antibody (mAb) for 72 h and treated or not with silibinin for the last 48 h of culture **(C,D)**. Results from representative female (upper panels) and male (lower panels) RA patients are shown **(A,C)**. Numbers reported represent the percentages of AV positive/PI negative (early apoptotic, bottom right quadrant) and AV positive/PI positive (late apoptotic or necrotic cells, top right quadrant). Data referred to both AV positive/PI negative and AV positive/PI positive cells are also reported as mean ± SD **(B,D)**. **(E,F)** Cell proliferation was evaluated by flow cytometry measuring Ki-67 nuclear antigen expression in T lymphocytes after activation with anti-CD3 mAb for 72 h and treatment with silibinin for the last 48 h of culture. Results from representative female (upper panels) and male (lower panels) RA patients are shown **(E)**. Data are also reported as mean ± SD **(F)**. *P < 0.05 versus untreated cells.

**Figure 6:**
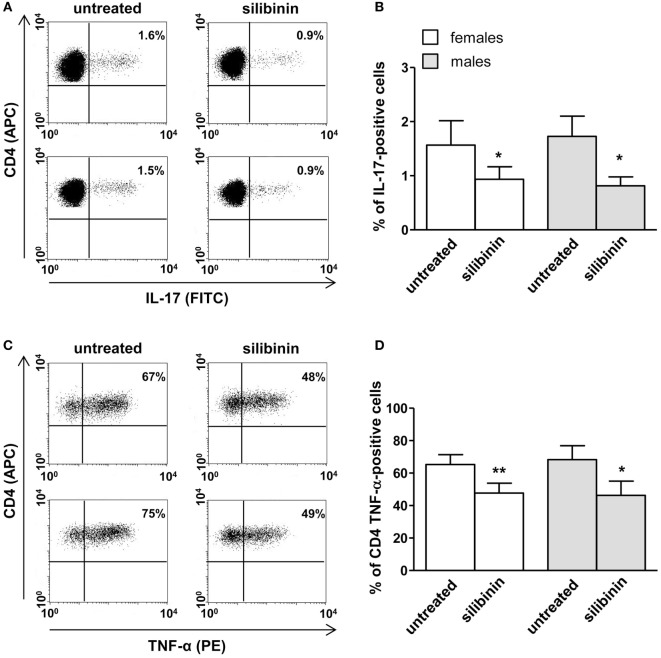
Silibinin-dependent effects on IL-17 and TNF-α expression in peripheral blood T lymphocytes from rheumatoid arthritis (RA) patients. T lymphocytes from 10 female and 4 male RA patients were evaluated by flow cytometry for cytokine production after 48 h of culture with silibinin and stimulation with phorbol myristate acetate (PMA) and ionomycin in the presence of brefeldin as detailed in Section “Materials and Methods.” Results from representative female (upper panels) and male (lower panels) RA patients are shown **(A,C)**. Data are also reported as mean ± SD **(B,D)**. *P < 0.05, **P < 0.01 versus untreated cells.

Based on the results obtained in healthy donors, we decided to test the anti-inflammatory potential of silibinin on T lymphocytes from female and male patients with active RA, analyzing its ability to modulate apoptosis, proliferation, and cytokine expression. Similarly to that observed in healthy donors, silibinin induced a significant increase of apoptosis in T lymphocytes from RA patients in both resting (*P* = 0.0161 for females and *P* = 0.0286 for males, Figures 5A,B) and activated state (*P* = 0.0403 for females and *P* = 0.0421 for males, Figures 5C,D). In addition, a significant reduction of proliferation level was detected after cell treatment with silibinin (*P* = 0.0160 for females and *P* = 0.0286 for males, Figures 5E,F). Notably, silibinin was able to significantly reduce IL-17 and TNF-α expression levels in CD4^+^ T lymphocytes (IL-17: *P* = 0.0235 for females and *P* = 0.0294 for males; TNF-α: *P* = 0.0032 for females and *P* = 0.0421 for males, versus untreated cells, respectively, Figures 6A–D). Similarly to that observed in healthy subject, no significant difference was observed between cells from male and female subjects after silibinin treatment.

### Silibinin Effect on miR-155 Expression in Peripheral T Lymphocytes From Patients With Active RA

**Figure 7:**
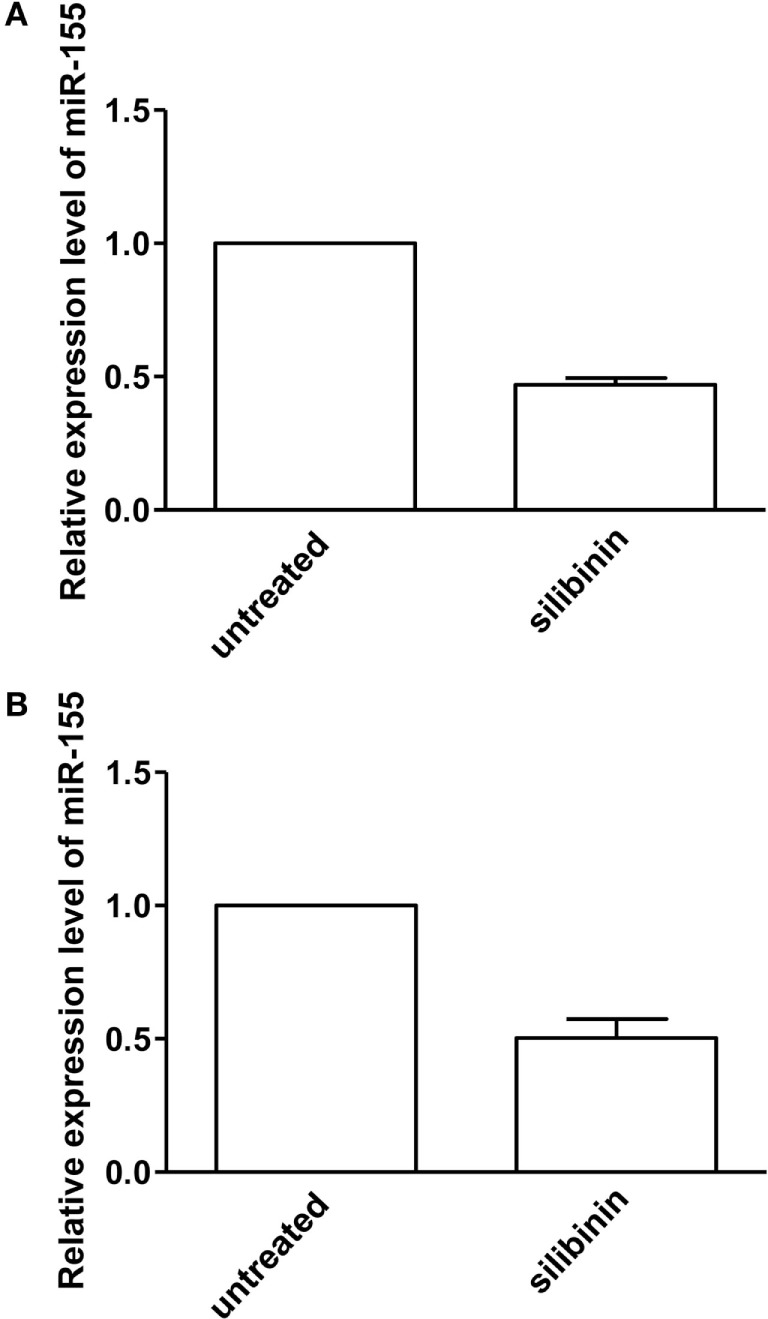
Quantitative real-time PCR (qRT-PCR) analysis of microRNA-155 (miR-155) expression level in peripheral blood T lymphocytes from rheumatoid arthritis (RA) patients. qRT-PCR analysis of miR-155-3p expression level in T lymphocytes isolated from five female **(A)** and two male **(B)** RA patients, treated or not with silibinin for 48 h. The values of fold change were calculated by the 2−ΔΔCt method relative to untreated RA patient. The mean ± SD values, referred to the fold of change between treated and untreated patients, were shown.

Finally, we asked whether silibinin could act as an epigenetic modifier modulating miRNA expression. We focused on miR-155 that plays a crucial role in the pathogenesis of RA (29–31) and which expression has been demonstrated to be modulated by estrogen through ERβ (38). Thus, we quantitatively analyzed miR-155 expression in T lymphocytes from RA patients, upon treatment with silibinin, by qRT-PCR assay. The results, shown in Figure 7, indicated an average 54 and 50% (for females and males respectively) decreased expression of miR-155 in T lymphocytes after silibinin treatment, thus suggesting that this phytoestrogen acted as a downregulator of miR-155.

## Discussion

Our study provides new insights regarding the anti-inflammatory effects of the phytoestrogen silibinin in T cell immunity. First, we demonstrated that silibinin upregulates ERβ expression, induces apoptosis, inhibits proliferation, and reduces expression of the pro-inflammatory cytokines IL-17 and TNF-α, through ERβ binding, in T lymphocytes from both female and male healthy subjects. Then, we confirmed these results in T lymphocytes from patients with RA in term of apoptosis, proliferation, and cytokine production. Finally, we found that silibinin acts as an epigenetic modifier, down-modulating the expression of miR-155 which plays a key role in the pathogenesis of RA.

Growing evidence suggests that ERα and ERβ subtypes mediate distinct transcriptional activities when they are co-expressed in the same cells and that the quantity and distribution of these receptors are crucial for their biological effects (8). In particular, ERβ displays an anti-inflammatory effect and the upregulation of this receptor in immune cells may provide a useful tool in creating an anti-inflammatory milieu. Here, we observed for the first time that silibinin was able to upregulate ERβ expression in T lymphocytes from both women and men.

Ligation of this receptor by silibinin induced an antiproliferative and a proapoptotic effect in T lymphocytes. These results are partially in accordance with those previously reported by other groups who observed that silymarin plays an antiproliferative activity (39–41) but, unlike what we have seen, it also has a proapoptotic effect (39, 40) in human T lymphocytes. The use by these authors of silymarin, which consists of a family of flavolignans including silybinin, isosilybinin, silychristin, isosilychristin, silydianin, and the flavonoid taxifoline, instead of the pure silibinin, might account for this discordant result. Interestingly, silibinin was able to strongly decrease the expression of the pro-inflammatory cytokines IL-17 and TNF-α through a mechanism that foresees its binding to ERβ, as demonstrated by the lack of silibinin effect when this receptor was knocked down. To note, all these experiments revealed that T lymphocytes from both sexes had the same susceptibility to silibinin. This is in line with previous observations that T lymphocytes from females and males subjects express similar basal levels of ERβ (12) and with our finding that silibinin upregulated ERβ in both sexes, assigning to silibinin a role for the treatment of inflammatory diseases both in women and in men. To note that the selective activation of ERβ could be of clinical value since it does not induce the classic side effects, mediated by ERα, observed after estrogen administration (e.g., cerebro- and cardiovascular events, higher occurrence of endometrial and breast cancer).

Both IL-17 and TNF-α are known to play a critical role in the pathogenesis of RA as demonstrated by the success of treatments based on their inhibition by biological disease-modifying antirheumatic drugs (42, 43). Interleukin-17 and TNF-α modulate the migration of inflammatory cells into the joints and display additive or synergistic effects on human synovial fibroblasts (44). Accordingly, alterations in peripheral T lymphocyte homeostasis and unbalanced Th1 and Th17 cytokine profiles have been repeatedly demonstrated in RA patients (27). Thus, a drug able to block these two cytokines at the same time would be very useful for an effective therapeutic approach in inflammatory arthritis. Hence, with in mind the goal to propose silibinin as therapeutic tool in RA, we evaluated whether this phytoestrogen could exert its anti-inflammatory effects on T lymphocytes from patients with active RA who had a poor response to disease-modifying antirheumatic drugs. In agreement with the results obtained in healthy donors, silibinin appeared to have immunosuppressive/anti-inflammatory effects on T lymphocytes from RA patients inducing apoptosis, inhibiting proliferation and both IL-17 and TNF-α expression, thus assigning to this molecule a potential value as therapeutic tool in this disease. In support of this assumption, a recent study by Tong et al. (45) showed that silibinin alleviated inflammation and induced apoptosis in human RA fibroblast-like synoviocytes and had a beneficial effect on arthritis in rats.

Interestingly, Th1 and Th17 polarization have been recently associated with an overexpression of miR-155 whose deregulation plays a crucial role in the pathogenesis of RA contributing to the progress of inflammation (29, 30). miR-155 has been reported to be abnormally expressed in arthritis models and miR-155-deficient mice do not develop collagen-induced arthritis and show significant reduced Th17 cells and autoantibody production (46). Furthermore, miR-155 expression in RA patients has been positively related to TNF-α, C-reactive protein, erythrocyte sedimentation rate levels, and DAS28 (47). Noteworthy, in this study, we observed a downregulation of miR-155 expression in T lymphocytes from RA patients treated with silibinin. Accordingly, He et al. (38) demonstrated that miR-155 is downregulated by estrogen through ERβ, further supporting the crucial role of this receptor as potential therapeutic target in RA.

In conclusion, our *in vitro* study provided new insights regarding the anti-inflammatory activities of silibinin. However, *in vivo* assays (e.g., collagen-induced arthritis model) are needed to confirm the potential role of this compound as therapeutic tool in RA, paving the way for clinical trials in this disease. In particular, the use of silibinin in combination with synthetic drugs might reduce their standard dosage and their related side effects. To note, clinical trials on silibinin effectiveness in the treatment of patients with hepatitis, cirrhosis, or biliary disorders demonstrated its safety with adverse events comparable to placebo (24, 48).

## Ethics Statement

Investigation has been conducted in accordance with the ethical standards and with the Declaration of Helsinki, and according to national and international guidelines. It was approved by the institutional review board of Policlinico Umberto I (Rome, Italy). All enrolled subjects were provided with complete information about the study and asked to sign an informed consent.

## Author Contributions

MD, MG, AM, AR, SA, AF, and MTP designed and performed *in vitro* experiments and analyzed data. MS performed flow cytometry and cell sorting analysis. CA and FCe contributed to patient enrollment and sample collection. FCo, LG, and GV provided intellectual input throughout the study. MP and EO provided important contribution to the conception of the work as well as interpretation of data and manuscript writing. All the authors read and approved the final manuscript.

## Conflict of Interest Statement

The research was conducted in the absence of any commercial or financial relationships that could be construed as a potential conflict of interest.

## Abbreviations

APC, allophycocyanin; AV, annexin V; DAS28, disease activity score 28; E2, 17β-estradiol; ER, estrogen receptor; FITC, fluorescein isothiocyanate; GAPDH, glyceraldehyde 3-phosphate dehydrogenase; mAb, monoclonal antibody; miR-155, microRNA-155; PBMC, peripheral blood mononuclear cell; PE, phycoerythrin; PHTPP, 4-[2-phenyl-5,7-bis(trifluoromethyl)pyrazolo[1,5-*a*]pyrimidin-3-yl]phenol; PI, propidium iodide; PMA, phorbol myristate acetate; RA, rheumatoid arthritis; qRT-PCR, quantitative real-time PCR; siRNA, small interfering RNA; SSNC, silencer select negative control.
