# Supplementary material for: The Natural Agonist of Estrogen Receptor β Silibinin Plays an Immunosuppressive Role Representing a Potential Therapeutic Tool in Rheumatoid Arthritis
Source: Front Immunol. 2018 Aug 17;9:1903. doi: 10.3389/fimmu.2018.01903 (PMC6107853; doi:10.3389/fimmu.2018.01903)
Supplement: Supplementary file 1 [file data_sheet_1.PDF]

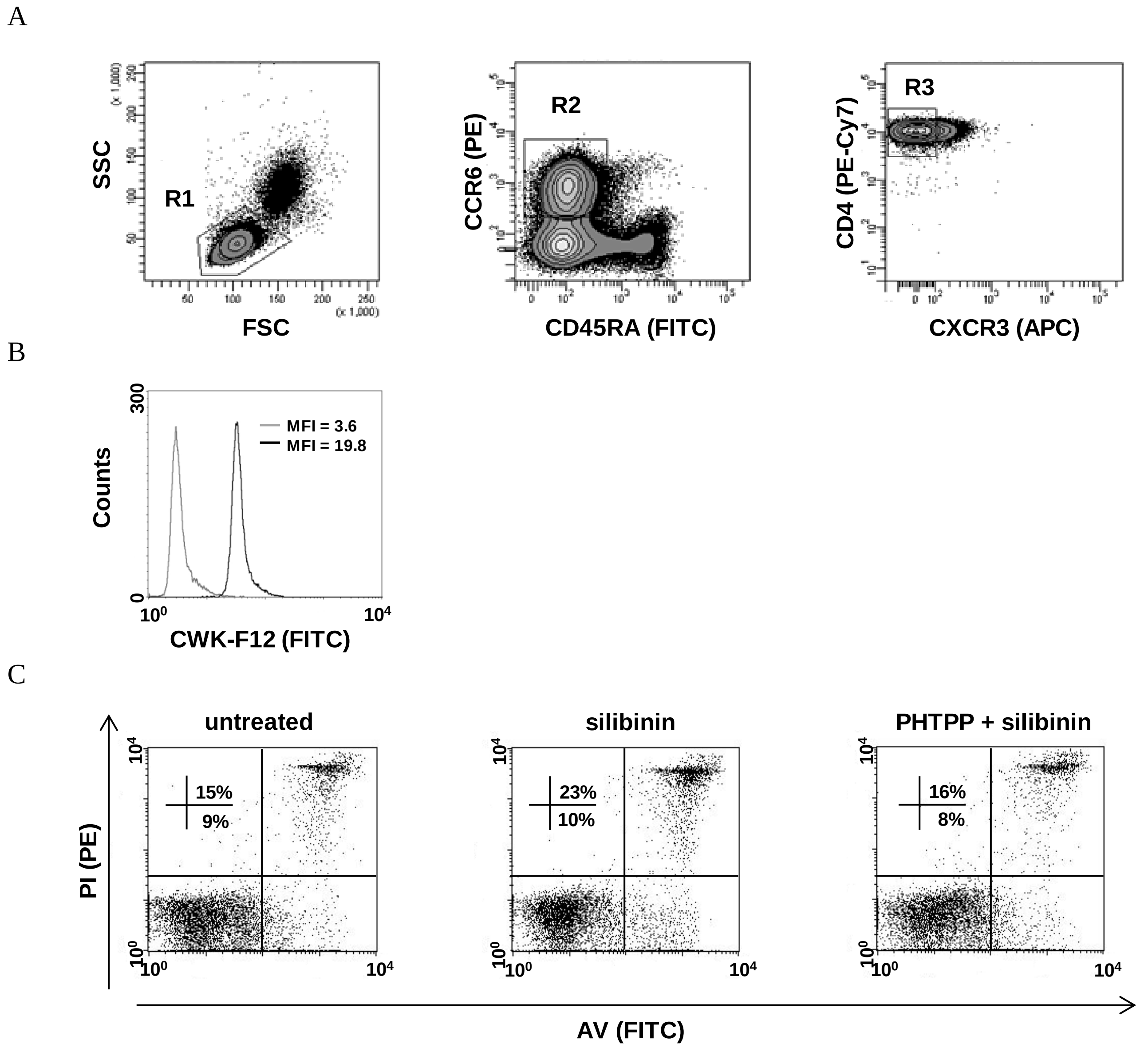


**Supplementary Figure 1**. ERβ expression and silibinin-dependent effects on apoptosis of Th17 lymphocytes from a representative healthy subject. (**A**) Gating strategy for cell sorting. Total CD4 T cells were isolated by positive selection using CD4 magnetic microbeads (Miltenyi Biotech). Memory Th17 cell subset was sorted to over 95% purity after gating on the basis of size and granularity (R1) and on $CD45RA^{-}CCR6^{+}$ (R2), and on $CXCR3^{-}$ (R3) cells. (**B**) Intracellular ERβ expression analysis was performed by flow cytometry in Th17 cells activated by anti-CD3 mAb for 72 h. Representative flow cytometry plot shows the fluorescence intensity of anti-ERβ mAb (clone CWK-F12) compared with isotype control. Isotype control staining is represented by the gray line and anti-ERβ mAb staining by the solid line. Values of the respective mean fluorescence intensity (MFI) are reported. (**C**) Apoptosis assay involving dual staining with AV and PI was carried out using flow cytometry in Th17 cells activated by anti-CD3 mAb for 72 h under the following conditions: i) mock (left panel); ii) silibinin treatment for the last 48 h of culture (middle panel); iii) pretreatment with the ERβ antagonist PHTTP 1h before adding silibinin (right panel). Numbers reported represent the percentages of AV positive/PI negative (early apoptotic, bottom right quadrant) and AV positive/PI positive (late apoptotic or necrotic cells, top right quadrant).
